# The Impact of Parental Presence on Invasive Procedures in the Pediatric Emergency Department: A Prospective Study

**DOI:** 10.3390/jcm12175527

**Published:** 2023-08-25

**Authors:** Saar Hashavya, Naama Pines-Shwartz, Noa Guzner, Lea Ohana Sarna Cahan, Itai Gross

**Affiliations:** 1 Department of Pediatric Emergency Medicine, Hadassah-Hebrew University Medical Center, Jerusalem 91120, Israel; saarha@gmail.com (S.H.); naamapines@gmail.com (N.P.-S.); lea810@gmail.com (L.O.S.C.); 2 Faculty of Medicine, Hadassah-Hebrew University Medical Center, Jerusalem 91120, Israel; noa.guzner@mail.huji.ac.il

**Keywords:** children, procedure, sedation, pediatric emergency department (PED)

## Abstract

Parental presence during invasive pediatric procedures is controversial, and its benefits are under-researched. The objective of this study was to assess the effects of parental presence during invasive procedures on the parents themselves and the physician performing the procedure. This prospective study was conducted at a single tertiary center in Jerusalem, Israel. During 10 shifts, all physicians and the families of patients who underwent invasive procedures in the pediatric emergency department (PED) were asked to fill in questionnaires related to their experiences. A total of 98 parental questionnaires and 101 physician questionnaires were collected. The most commonly performed procedures were laceration repair (65%) and abscess drainage (18%). Sedation was required in 75% of cases. In total, 73% of the cited family members were present during these procedures. The main reason for refusing to allow family members access was the physicians’ concern that the procedure would be hard for parents to watch. However, in more than 85% of cases, the physicians felt that the presence of a family member contributed to the success of the procedure, augmented the child’s sense of safety and lessened the family members’ feelings of anxiety. All parents who opted to be present during the procedure felt very satisfied, compared to 67% of parents who were not present (*p* < 0.0001). When asked if, in retrospect, they would have made the same decision, 100% of the parents who were present during the procedure indicated that they would have made the same decision, compared to only 68% of the parents who were not present (*p* < 0.001). Overall, these findings highlight the positive effects of parental presence during invasive procedures performed in the PED, even when procedures were performed under sedation. Encouraging parental attendance during invasive procedures may, thus, enhance family-centered practices in the PED.

## 1. Introduction

Parental presence during invasive pediatric procedures is controversial, and its benefits are under-researched. Traditionally, parents are asked to remain outside of the procedure room or operating theater. However, with the growth of family-centered care, family input into medical decisions has increased, and the traditional approach has accordingly changed [1,2,3,4]. Studies suggest that most families want to be involved in their child’s healthcare and medical decision-making [5,6,7]. The implications of this trend have attracted considerable research attention, and a variety of studies have compared different attitudes to and aspects of the subject [6,8,9].

The findings show that family members are often not allowed to be present in the operating room to support their child during an invasive procedure [8]. For a variety of reasons, healthcare professionals are reluctant to allow parents to participate in invasive procedures, with justifications ranging from tradition to the belief that the parent will interfere with the procedure [1,9] (specifically that the parental presence may undermine the medical teams’ ability to freely make differential diagnoses or allow interns and residents to carry out certain parts of the procedure). However, parental presence may reduce the anxiety thresholds of both the patient and the family members themselves, which results in better cooperation [5,10,11]. A calm and supportive presence is more likely to enable children to adaptively respond to stress. A trusted family member can alleviate fear and anxiety by providing comfort, guidance and encouragement [1,2,5,9,12].

One randomized controlled trial showed that children’s level of pain significantly decreased when their parents were present during the administration of infusions or injections compared to children whose parents were not present [10]. In another study, surveys were used to compare the anxiety levels of parents who were present during their child’s intensive care unit procedures to those of parents who were not permitted to be present. The children’s and parents’ anxiety levels significantly declined when parents were in attendance. In addition, 94% of the nursing staff indicated that parental presence was beneficial to both the child and the parents [11].

Current research supports recommendations to allow family members to be present during procedures and resuscitation. In the United States, the American Academy of Pediatrics and the American College of Emergency Physicians also encourage parental presence during invasive procedures [3]. Nevertheless, there are a lack of policies, guidelines or documentation protocols in most hospitals [5,9]. In Israel, there is no official policy regarding the presence of family members during invasive procedures in pediatric emergency medicine (PED), with the most common practice being that in cases in which the patient is awake, the parents are encouraged to attend the procedure, but when the patient is sedated, they are asked to step outside. The objective of our study was to assess the effect of parental presence on the parents themselves and the physician performing the procedure. In our PED, a variety of invasive procedures are carried out on a daily basis, some of which occur under sedation. In most cases, when the patient is sedated, the parents are asked to step outside of the procedure room. In this study, parents completed a questionnaire related to their feelings and anxiety levels both when present while their children underwent invasive procedures in the PED or when not in attendance. The physician performing the procedure also completed a questionnaire. 

## 2. Methods

### 2.1. Sample

The study was prospectively conducted at the Hadassah Ein Kerem Medical Center, Jerusalem, Israel, from February to March 2023. This study was approved by the Institutional Review Board (approval #HMO—0441-22). During 10 shifts, families of all patients who underwent invasive procedures in the PED were asked to fill out questionnaires after the procedure was completed (Supplementary Material Questionnaire S1) based on their experiences before, during and after the procedure. The physician who performed the procedure was also asked to fill in a questionnaire (Supplementary Material Questionnaire S2). 

There was no randomization of the interventions as a function of parental presence during the procedure. For each case, the decision to attend was made by the physician and the parents. The questionnaires were administered in instances of invasive procedures including suturing, abscess drainage, burn treatments, lumbar puncture and joint and fracture reduction. 

Sedation involved the administration of nitrous oxide, midazolam, propofol or ketamine. All procedures were conducted in the PED procedure rooms. 

Parents’ and physician’s questionnaires regarding patients who underwent invasive procedures in the PED were included. Patients who underwent the procedures in the operating theater were excluded. Incomplete questionaries with missing critical information were excluded from the data analysis. 

### 2.2. Data Collection

During 10 shifts, following every invasive procedure, the attending pediatric emergency medicine physician asked the patients’ family members and the physician performing the procedure to fill in a questionnaire (Supplementary Material Questionnaires S1 and S2). In cases in which more than one family member was present, each family member was asked to fill out the questionnaire. Study questionnaires used a 5-point Likert scale. The questionnaires were designed for this study and included information regarding the type of procedure, whether sedation was used and the family member who was in attendance (Mother/Father/Other). Parents were asked to state their reasons for wanting to be present or to not attend the procedure, as well as their state of anxiety before, during and after the procedure. They were also asked whether, in retrospect, they would have taken the same course of action.

The physicians who performed the procedure were asked to indicate their subspecialty, the type of procedure and whether sedation was required. They were also asked to state their reasons for permitting or refusing to allow parental presence during the procedure, their views as to how the parental presence affected the course of the intervention and whether they would have made a different decision in retrospect. 

### 2.3. Statistical Analysis

The data were presented as numbers and percentages. The chi-square test was used to compare proportions. All *p*-values were two tailed and were considered to be statistically significant at *p* ≤ 0.05. Statistical analysis was performed using SPSS version 21.0 (Statistical Package for Social Science, Chicago, IL, USA). 

Sample size calculations were carefully considered to ensure the study’s robustness. The calculation aimed to detect statistically significant differences in the reported emotional changes before and after invasive medical procedures were performed based on attendance status, with an alpha level of 0.05. Based on preliminary data and a desired statistical power of 80%, the final sample size was determined to be at least 80 participants. Ten consecutive shifts were used to collect the questionnaires.

## 3. Results

A total of 98 parental questionnaires and 101 physician questionaries were collected. Four parents and none of the physicians refused to fill in the questionnaires. Twelve questionnaires were incomplete and excluded. Of the family members accompanying the patients, 45 were mothers (46%), 44 were fathers (45%) and the remaining individuals were other family members. The most common procedures were laceration suturing (65% of cases) and abscess drainage (18% of cases). Sedation was required in 75% of the cases. 

### 3.1. Physicians’ Questionnaires

As shown in Table 1, 76% of the physicians asked the parents if they wanted to be present during the procedure, and 73% of parents accepted that offer. The primary reason for refusing to allow parents to attend the procedure was the physician’s concern that the procedure would be hard to watch (11/19; 58%). The next most frequent reason for forbidding parental presence was the supervision of interns/residents, which accounted for 4/19 (21%) of the cases.

In cases in which the family members attended the procedure, the physicians reported that 76% were actively involved in the procedure (holding/comforting/distracting), and in 95% of cases, they were able to explain and educate the parents during the procedure. In 86% of cases, the physicians reported that parental presence was very or extremely helpful in terms of contributing to the success of the procedure. Similarly, in 87% of cases, they felt it was very or extremely helpful in managing the child’s behavior, and in 93% of cases, they felt it was very or extremely helpful in lessening the family members’ anxiety. 

In cases in which the parents were present, the physicians reported that 89% of procedures were extremely successful, compared to 62% of cases in which the parents were not in attendance (*p* < 0.01). When parents attended the procedure, 98% of the physicians reported that, in retrospect, they would have maintained their decision to allow the parents to stay in the procedure room, compared to 45% of the physicians who did not allow the parents to attend the procedure (*p* < 0.0001). 

### 3.2. Parents’ Questionnaire 

As shown in Table 2, 77% of parents reported that they had been invited by the physician to attend the procedure. Of these parents, 69% chose to be present. The main reason that parents did not attend the procedure was their own decision not to attend (68%), and the rest (32%) of the group were willing to be present but were forbidden from attending by the physician. When the procedure was conducted under sedation, 53% of parents were present. 

As shown in Table 2, 78% of parents felt that they played an active role during the procedure, and 94% of parents felt that their presence was very or extremely helpful to their child. Parents who attended the procedure felt extremely satisfied in with the pre-procedure explanation 89% of cases, compared to 60% of parents who did not attend (*p* < 0.001); 97% of parents were very or extremely satisfied with the explanation given during the procedure. 

Of the parents who were present during the procedure, 40% felt very or extremely anxious before the procedure, compared to 77% of parents who did not attend the procedure (*p* < 0.001). There was no difference between groups in terms of anxiety reduction post-procedure (68% vs. 78%; *p* = 0.36). 

All parents who were present during the procedure felt very or extremely satisfied, compared to 67% of parents who did not attend the procedure (*p* < 0.0001). When asked if, in retrospect, they would have made the same decision, 100% of parents who were present during the procedure indicated that they would have maintained their choice, compared to 68% of parents who did not attend the procedure (*p* < 0.001).

## 4. Discussion 

This prospective study examined parental presence during invasive pediatric procedures in the PED. The findings show that parental attendance was associated with higher satisfaction and a greater likelihood of attending procedures in the future, if permitted. The physicians perceived parental presence as beneficial to the success of procedure, child comfort and parental wellbeing. 

Previous studies carried out in the pediatric intensive care unit (PICU) setting have reported greater familial satisfaction and decreased anxiety when the parents are present during invasive procedures and even resuscitation [11,13,14]. Nevertheless, in many medical centers, parental presence during invasive procedures, particularly those involving sedation or anesthesia, are not encouraged and, at times, are not allowed [15,16]. 

The emergency department is known to be a highly stressful setting for patient interactions with the health care system due to the acute nature of the referral, the crowded environment and the lack of familiarity with the attending physicians and staff. All of these factors increase levels of anxiety and fear in both the patient and their family [1]. 

In studies conducted in the emergency department, parental involvement in decision-making and during venipuncture was found to reduce levels of anxiety and increase overall parent and child satisfaction [14,16]. In the current study, parental presence during the procedure and parental invitations to attend the procedure were high compared to other studies, which reported 10–40% attendance rates [16]. The findings here are in line with the emergency department policy that promotes a family-centered approach.

The findings showed that parental presence during invasive procedures, such as lacerations, suturing and abscess drainage, led to an increased parental sense of satisfaction and a feeling of greater competence. All of the parents who were present during the procedures reported that they were happy with their decision, compared to 68% of those who did not attend. Similar observations have been noted in other setups, such as in the PICU, NICU and post-surgical recovery units [11,16], thus emphasizing the importance of parental presence during invasive procedures.

Consistent with reports, as the complexity of the procedure increases, the rate of parental presence decreases [16]. In the current study, family members were present in only 53% of the procedures conducted under sedation, compared to 100% of the cases in which the patient was awake. It is obvious that in cases where deep or dissociative sedation is administered, the presence of a family member is less crucial for the patient. As shown here, however, it was considered beneficial for the family members. 

Interestingly, out of the 31% of parents who were not present during the procedure, only one third were prevented by the physician from attending, suggesting that improved communication and information sharing prior to the procedure might be beneficial and increase physicians’ willingness to authorize parental presence. 

Similar to reports in the literature, parental anxiety was the most common reason expressed by physicians who decided not to permit parents to attend a procedure [17]. The anxiety level after a procedure was significantly higher in parents who were not present, with 48.1% of these parents reporting very or extremely high anxiety, compared to 15.6% of the parents who attended the procedure (*p* = 0.001). As the reported anxiety level in this group was also higher before the procedure, the data analysis failed to show a significant difference in the reduction in anxiety levels between groups (*p* = 0.36). 

Intern/resident training and the concomitant fear of procedure failure due to parental presence were the other most common concerns among physicians. One study found more reluctance to authorize parental attendance among junior staff [11,16].

Nevertheless, when parents were present during the procedure, the physicians reported higher success rates and indicated that, in retrospect, they would have made the same decision to authorize parental presence, thus emphasizing the importance of parental presence in the eyes of physicians. 

## 5. Limitations

This study has a number of limitations. The first limitation is the lack of randomization, which may have resulted in a response bias toward parents and physicians who were family practice oriented or parents who were more prepared and capable of self-control and, thus, chose to attend the procedure. The second limitation is social desirability bias, which may have led to more positive responses, although the questionnaires were anonymous. In addition, the physicians were aware that we were conducting a study of parental presence during procedures and might have decided to allow more parents to attend. We, however, believe that this issue did not significantly alter the conclusions of this study. Finally, this study was conducted in a single center in Israel; hence, caution should be exercised when extending these conclusions to other centers and countries. 

In conclusion, this study highlights the beneficial effects of parental presence during invasive procedures in the PED, even when performed under sedation. Both parental reluctance and the treating physicians’ fear of parental anxiety were the main reasons for parental absence during the procedures. Formulating an intervention program to increase parental presence during procedures and refuting misguided fears might help to increase family-centered practices in the pediatric emergency department.

## Figures and Tables

**Table 1:** Physicians’ questionnaire. Comparison between cases in which a family member of the patient either attended or did not attend the procedure in the pediatric emergency department.

	Parent Present N (%)	Parent Not Present N (%)
Procedure	N = 74	N = 27
Suture	56 (76)	10 (37)
Abscess drainage	10 (14)	8 (30)
Burn treatment	3 (4)	3 (11)
Dislocation reduction	2 (3)	1 (4)
Foreign body removal	1 (1)	3 (11)
Lumbar puncture	1 (1)	2 (7)
IV insertion	1 (1)	
Sedation	N = 72 42 (58)	N = 20 20 (83)
Did the parents ask to be present during the procedure?	N = 70 60 (86)	N = 25 10 (40)
Did you give the parents the opportunity to attend the procedure?	N = 74 71 (96)	N = 27 6 (22)
If not, why not?		N = 19
I was worried they would find it difficult to watch.		11 (58)
I was concerned that the parents might prevent me from performing the procedure.		1 (5)
The procedure was part of intern/resident training.		4 (21)
This is standard protocol.		2 (11)
Other		1 (5)
Were the parents actively involved?	N = 74 56 (76)	
During the procedure, did you provide explanations?	N = 74 70 (95)	
To what extent do you think that parental presence contributed to the success of the procedure?	N = 73	
No contribution	0(0)	
Somewhat helpful	4 (6)	
Moderately helpful	6 (8)	
Very helpful	8 (11)	
Extremely helpful	55 (75)	
To what extent do you think that parental presence contributed to the child’s cooperation, level of anxiety and behavior?	N = 53	
No contribution	1 (2)	
Somewhat helpful	3 (6)	
Moderately helpful	3 (6)	
Very helpful	6 (11)	
Extremely helpful	40 (75)	
To what extent do you think that parental presence contributed to the management of parents’ own feelings?	N = 53	
No contribution	0 (0)	
Somewhat helpful	0 (0)	
Moderately helpful	4 (8)	
Very helpful	7 (13)	
Extremely helpful	42 (79)	
To what extent were you satisfied with the way the procedure was performed?	N = 74	N = 26
Not satisfied	0 (0)	1 (4)
Somewhat satisfied	0(0)	0 (0)
Moderately satisfied	3 (4)	0 (0)
Very satisfied	5 (7)	9 (35)
Extremely satisfied	66 (89)	16 (62)
In retrospect, would you have made the same decision to allow/refuse parental attendance ?	N = 52 51 (98)	N = 20 9 (45)

N = Total number of physicians answering each question.

**Table 2:** Parents’ questionnaire. Comparison between cases in which a family member of the patient either attended or did not attend the procedure in the pediatric emergency department.

	Present N (%) N = 68	Not Present N (%) N = 30	*p* Value
Procedure	N = 53	N = 22	
Suture	40 (75)	9 (40)	
Dislocation reduction	1 (2)	1 (5)	
Abscess drainage	8 (15)	6 (26)	
Lumbar puncture	1 (2)	1 (5)	
Burn treatment	1 (2)	1 (5)	
Foreign body removal	1 (2)	3 (14)	
Other	1 (2)	1 (5)	
Sedation	N = 51 27 (53)	N = 23 22 (96)	
Did the physician ask you to attend the procedure?	N = 64 62 (97)	N = 29 10 (35)	
If you were not present, why did you not attend?	N = 64	N = 25	
I wanted to be present in the room, but I was not permitted to attend.		8 (32)	
I was not permitted to be present in the room, and I did not want to attend.		5 (20)	
I did not want to be present during the procedure.		12 (48)	
If you were present, were you actively involved in the procedure?	N = 64 50 (78)		
To what extent were you satisfied with the explanation you received about the procedure before it was carried out?	N = 66	N = 30	
Not satisfied	1 (2)	0(0)	
Somewhat satisfied	0 (0)	1 (3)	
Moderately satisfied	0 (0)	6 (20)	
Very satisfied	6 (9)	5 (17)	0.28
Extremely satisfied	59 (89)	18 (60)	0.0009
To what extent were you satisfied with the explanation you received while the procedure was being performed?	N = 65		
Not satisfied	1 (2)		
Somewhat satisfied	0(0)		
Moderately satisfied	1 (2)		
Very satisfied	5 (8)		
Extremely satisfied	58 (88)		
To what extent do you believe that your presence in the room helped your child to feel safe?	N = 63		
No effect	1 (1)		
Somewhat helpful	0(0)		
Moderately helpful	3 (5)		
Very helpful	8 (13)		
Extremely helpful	51 (81)		
How anxious were you before the procedure?	N = 65	N = 30	
Not anxious	8 (12)	1 (3)	0.17
Minimally anxious	15 (23)	1 (3)	0.012
Moderately anxious	16 (25)	5 (16)	0.39
Very anxious	9 (14)	7 (23)	0.25
Extremely anxious	17 (26)	16 (53)	0.01
How anxious were you after the procedure?	N = 64	N = 27	
Not anxious	33 (51)	1 (4)	*p* < 0.0001
Minimally anxious	14 (22)	5 (18)	0.72
Moderately anxious	7 (11)	8 (30)	0.03
Very anxious	5 (8)	9 (33)	0.002
Extremely anxious	5 (8)	4 (15)	0.31
Drop in stress level	N = 63 43 (68)	N = 27 21 (78)	0.36
In retrospect, would you have made the same decision?	N = 38 38 (100)	N = 22 15 (68)	0.0002
To what extent were you satisfied with the way in which the procedure was performed?	N = 67	N = 30	
Not satisfied	0 (0)	0 (0)	
Somewhat satisfied	0 (0)	2 (7)	
Moderately satisfied	0 (0)	8 (26)	
Very satisfied	8 (12)	2 (7)	0.44
Extremely satisfied	59 (88)	18 (60)	0.0016

N = Total family members answering this specific question.

## Data Availability

Data is available following a written request to itaig@hadassah.org.il.

## Supplementary Materials

The following supporting information can be downloaded at: https://www.mdpi.com/article/10.3390/jcm12175527/s1, Questionnaire S1. Parents questionnaire. Questionnaire S2. Physician questionnaire.

## Abbreviation

PED—Pediatric emergency department.
