# Supplementary material for: The Impact of Parental Presence on Invasive Procedures in the Pediatric Emergency Department: A Prospective Study
Source: J Clin Med. 2023 Aug 25;12(17):5527. doi: 10.3390/jcm12175527 (PMC10487884; doi:10.3390/jcm12175527)
Supplement: Supplementary file 1 [file jcm-12-05527-s001.zip › jcm-2535532-supplementary.pdf]

Supplementary files

Questionnaire S1

Parents' Questionnaire

1. **Type pf procedure**: ________________
2. **Sedation** Yes/no
3. **Did the physician ask you whether you wanted to be present during the procedure?** Yes/no
4. **Why weren't you in the procedure room?**
   a. I wanted to be present, but I wasn't permitted.
   b. I was not permitted, and I didn't want to.
   c. I didn't want to be present during the procedure.
5. **Were you actively involved during the procedure?** Yes/no
6. **To what extent were you satisfied with the explanation you received about the procedure before it was carried out?**
   a. Poorly satisfied
   b. Somewhat satisfied
   c. Moderately satisfied
   d. Very satisfied
   e. Extremely satisfied
7. **To what extent were you satisfied with the explanation you received while the procedure was being performed?**
   a. Poorly satisfied
   b. Somewhat satisfied
   c. Moderately satisfied
   d. Very satisfied
   e. Extremely satisfied
8. **To what extent do you believe that your presence in the room helped your child feel safe?**
   a. No effect
   b. Somewhat helpful
   c. Moderately helpful
   d. Very helpful
   e. Extremely helpful

9. **How much anxiety did you experience before the procedure?**
    a. None
    b. Minimally anxious
    c. Moderately anxious
    d. Very anxious
    e. Extremely anxious
10. **How much anxiety did you experience after the procedure**?
    a. None
    b. Minimally anxious
    c. Moderately anxious
    d. Very anxious
    e. Extremely anxious
11. **In retrospect, if you could have changed your decision, would you have done so?**
    Yes/no
12. **To what extent were you satisfied with the way the procedure was performed?**
    a. Not satisfied
    b. Somewhat satisfied
    c. Moderately satisfied
    d. Very satisfied
    e. Extremely satisfied

Questionnaire S2

Physicians' Questionnaire

1. **Type of Procedure**: ________________
2. **Sedation** Yes/no
3. **Did the parents ask to be present during the procedure?** Yes/no
4. **Did you give the parents the opportunity to be present during the procedure?** Yes/no
5. **If you objected to parental presence, what motivated your decision?**
    a. I was worried they would find it difficult to watch.
    b. I was concerned that the parents might prevent me from performing the procedure.
    c. I was teaching an intern/resident/junior staff and felt that parental presence would be an additional stressor.
    d. This is standard protocol.
    e. Other (please specify).
6. **Were they actively involved during their presence?** Yes/no
7. **During the procedure, did you provide explanations?** Yes/no
8. **To what extent do you think the parents' presence contributed to the success of the procedure?**
    a. No contribution
    b. Somewhat helpful
    c. Moderately helpful
    d. Very helpful
    e. Extremely helpful
9. **To what extent do you think the parents' presence contributed to the child?**
    a. No contribution
    b. Somewhat helpful
    c. Moderately helpful
    d. Very helpful
    e. Extremely helpful
10. **To what extent do you think the parents' presence contributed to their own feelings?**
    a. No effect
    b. Somewhat helpful

c. Moderately helpful
d. Very helpful
e. Extremely helpful

11. **To what extent are you satisfied with the way the procedure was performed?**
    a. Poorly satisfied
    b. Somewhat satisfied
    c. Moderately satisfied
    d. Very satisfied
    e. Extremely satisfied
12. **In retrospect, would you have changed your mind about parental presence based on the outcome if you had the chance?** Yes/no
